# Clinical Application of Suture Distraction Technique for Closure of Decompression Incisions Following Lower-Leg Compartment Syndrome: A Retrospective Cohort Study

**DOI:** 10.3390/jcm15207971

**Published:** 2026-10-15

**Authors:** Yuan Pan, Tao Wang, Min An, Yingying Deng, Chao Feng

**Affiliations:** 1 Beijing Jishuitan Hospital Guizhou Hospital, Guiyang 550014, China; sdpy16@163.com (Y.P.);; 2 Beijing Jishuitan Hospital, Capital Medical University, Beijing 100035, China

**Keywords:** suture distraction technique, acute compartment syndrome, decompression incision, polydioxanone suture, skin graft-free

## Abstract

**Objectives:** The objective of this study was to assess the suture distraction technique (SDT) for closing decompression wounds resulting from acute compartment syndrome (ACS) of the lower leg, and to examine whether it may serve as an alternative to skin grafting (SG). **Methods:** We retrospectively reviewed 67 patients who received decompressive fasciotomy for lower-leg ACS from January 2018 to June 2025. Of these, 31 were managed with SDT and 36 with SG. Wound healing time served as the primary outcome; secondary outcomes comprised the number of operations, hospital stay, wound-closure-related costs, sensory function, Vancouver Scar Scale (VSS) scores, donor-site injury, and infection rate. **Results:** The two groups were broadly similar in baseline characteristics. Compared with the SG group, the SDT group required fewer operations (median 1.0 vs. 2.0), achieved faster wound healing (14.0 vs. 18.5 days), had shorter hospital stays (27.0 vs. 46.0 days), and incurred lower wound-closure-related costs (1656 vs. 14,926.5 CNY) (all *p* < 0.001). VSS scores favored the SDT group (0.0 vs. 4.0, *p* < 0.001). All SDT patients preserved normal sensation, whereas all SG patients experienced hypoesthesia (*p* < 0.001). As an inherent consequence of skin grafting, every grafted patient had a donor-site wound, whereas no SDT patient required grafting and therefore had none; donor-site morbidity was described descriptively rather than compared statistically. The infection rate did not differ significantly between groups (*p* = 0.495); however, given the small number of events, no firm conclusion could be drawn. In the parsimonious adjusted analysis, the SG group had a significantly longer wound healing time (adjusted ratio, 1.412; 95% CI, 1.303 to 1.531; *p* < 0.001). The time from injury to fasciotomy was significantly longer in the SG group than in the SDT group (median 5.7 vs. 4.5 h; *p* = 0.012). **Conclusions:** Within this single-center retrospective cohort, SDT was linked to fewer operations, shorter wound healing time and hospital stay, and reduced costs, while preserving sensation and avoiding donor-site morbidity. As the decompression incision in ACS involves no actual loss of skin, this wound type may be particularly well suited to SDT, which enabled complete closure without skin grafting in the present cohort. These findings merit confirmation through prospective comparative studies.

## 1. Introduction

Acute compartment syndrome (ACS) of the lower leg arises when elevated pressure within an osteofascial compartment, enclosed by bone, the interosseous membrane, intermuscular septa, and deep fascia, compromises tissue perfusion and causes ischemic injury to muscles and nerves. Emergency decompressive fasciotomy remains the cornerstone of ACS management. After fasciotomy, however, the skin edges can seldom be re-approximated directly because of excessive wound tension, and many such wounds ultimately require skin grafting or other reconstructive procedures for definitive closure [1,2,3].

Skin grafting is a widely accepted means of reconstructing soft-tissue defects [2,3]; nevertheless, it inevitably introduces a donor-site wound that carries its own risks, including delayed healing, infection, unsatisfactory cosmesis, and superficial sensory disturbance, which together add considerably to the physical and psychological burden borne by patients.

A range of skin distraction devices and techniques has been explored worldwide to achieve primary or delayed closure of fasciotomy wounds. Shang et al. [4] obtained satisfactory results with pre-placed approximation sutures for delayed closure of decompression incisions. Wei et al. [5] and Lu et al. [6] introduced skin distraction devices enabling secondary wound closure after resolution of limb edema, with favorable outcomes; however, these methods entail an additional surgical procedure, and the specialized devices have not gained widespread use. Zhang et al. [7] applied anchored (fishbone-type) sutures for progressive skin approximation after fasciotomy and achieved early closure, although the non-sliding design may weaken the decompressive effect. Chen et al. [8] found both Nice knots and commercial distraction devices effective for small-to-medium soft-tissue defects, but the Nice knot technique proved relatively complex and delivered uneven tension. Song et al. [9] reported satisfactory closure of lower-leg and ankle wounds using an adjustable-tension distraction device, yet the associated costs and equipment requirements remain limitations.

Drawing on the Ilizarov tension–stress principle and the biomechanical behavior of extensible skin [10], we devised a progressive closure technique for ACS decompression incisions using polydioxanone (PDS) sutures, which yielded encouraging early results in our practice. This report summarizes our experience with the technique, and the study is reported in accordance with the Strengthening the Reporting of Observational Studies in Epidemiology (STROBE) checklist for cohort studies.

## 2. Materials and Methods

### 2.1. Patient Demographics and Clinical Data

**Figure 1:**
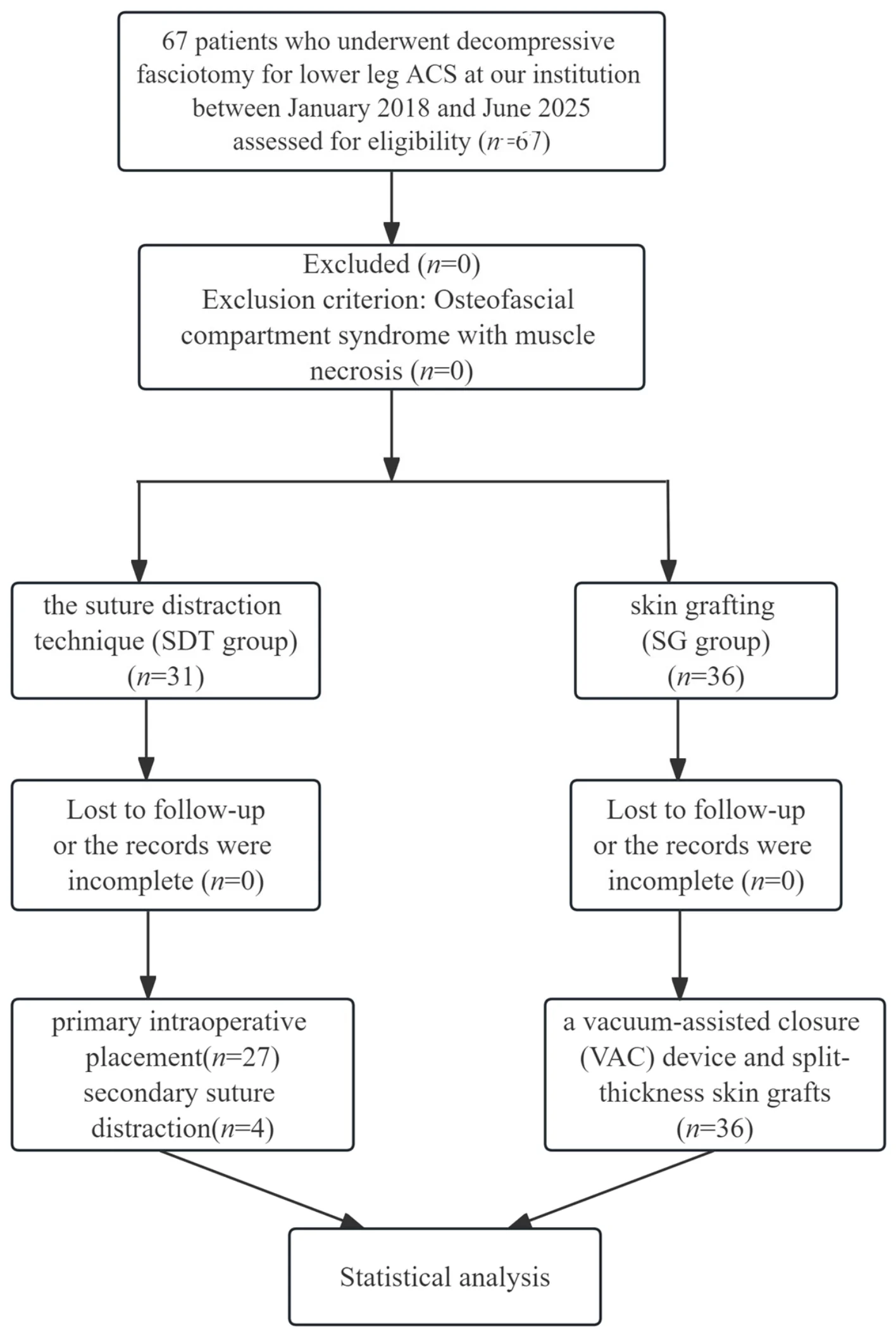
Patient enrollment flowchart.

Patients were enrolled consecutively according to the following inclusion criteria: (1) a clinical diagnosis of lower-leg ACS requiring emergency decompressive fasciotomy, established by characteristic findings (pain disproportionate to the injury, pain on passive stretch, tense compartments, and sensory or motor deficits) and, when available, compartment pressure measurement; (2) decompression incisions that required definitive wound closure; and (3) complete medical records with follow-up through wound healing. Patients were excluded if they had compartment syndrome with muscle necrosis, if their records were incomplete, or if they were lost to follow-up before wound healing. All patients undergoing decompressive fasciotomy for lower-leg ACS at our institution during the study period were considered for inclusion, and none were excluded for incomplete data. No SDT patient required conversion to skin grafting, and all SDT wounds closed completely as intended. Thus, no patient in whom SDT was initiated eventually required skin grafting and was therefore included in the SG group. Allocation to SDT or SG reflected the treating surgeon’s preference and the wound condition rather than a prespecified randomization schedule. The patient enrollment flowchart is presented in Figure 1.

**Figure 2:**
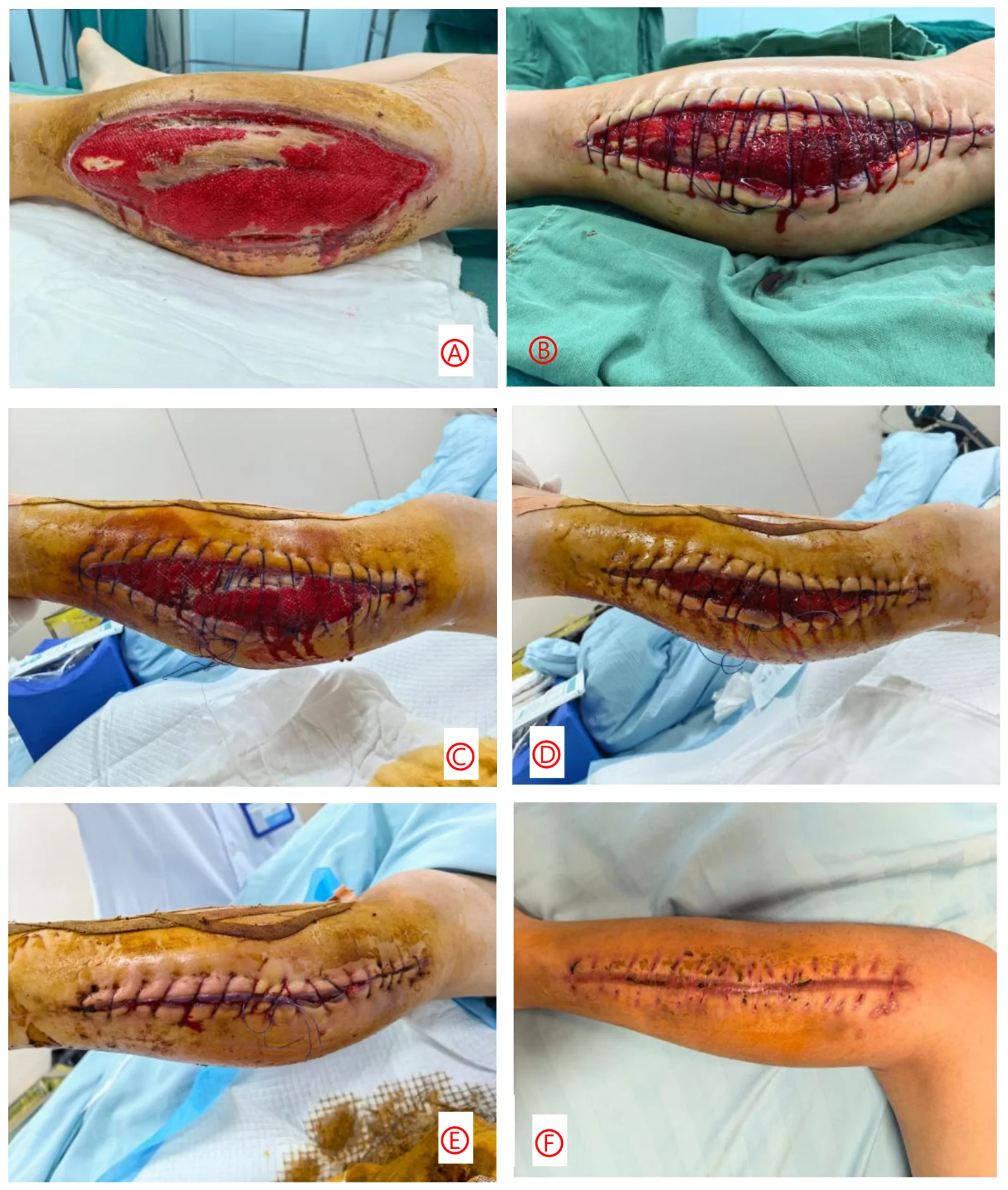
Closure of a unilateral fasciotomy incision for compartment syndrome with PDS suture distraction (secondary suture distraction). (**A**) Postoperative appearance of the fasciotomy incision; (**B**) appearance following surgical debridement with PDS suture distraction sutures placed; (**C**) Appearance after the first dressing change with suture tightening; (**D**) appearance after the second dressing change with suture tightening; (**E**) appearance after the third dressing change, demonstrating skin edge approximation; (**F**) appearance following skin edge approximation, with sutures removed.

Between January 2018 and June 2025, 67 patients who underwent decompressive fasciotomy for lower-leg ACS at our institution were identified and reviewed retrospectively. The cohort included 32 males and 35 females, with a mean age of 42.94 ± 14.76 years (range, 16–74 years). One patient had a single lateral decompression incision; the remaining 66 patients had dual medial and lateral incisions. Thirty-one patients received SDT, of whom 27 had distraction sutures placed primarily during the index fasciotomy and 4 underwent secondary suture distraction at a subsequent procedure. These four patients initially received conservative wound care with temporary negative-pressure wound therapy, because their wounds were not considered suitable for immediate suture placement at the index fasciotomy; once wound conditions permitted, they underwent secondary placement of distraction sutures at a later procedure and were classified in the SDT group according to their definitive closure method. For these four patients, wound healing time was likewise counted from the index fasciotomy to complete epithelialization, in accordance with the definition applied throughout this study. The other 36 patients underwent skin grafting (SG group). Notably, 4 patients in the SDT group and 6 in the SG group had previously been included in our earlier report on the suture-stretching technique for lower-limb skin defects [11]. All clinical photographs in this study were taken from the present cohort with written informed consent (Figure 2).

For both groups, we recorded wound dimensions (length and width), number of operations, wound healing time, hospital stay, surgical costs, and the number of suture tightening sessions. Table 1 summarizes the baseline characteristics.

Sensory function was tested with light touch and pinprick, and scars were scored with the Vancouver Scar Scale (VSS) at the final outpatient visit after wound healing, that is, at the last scheduled review performed after complete wound healing. A single trained assessor performed all evaluations; blinding to treatment allocation was not feasible because the presence or absence of a donor site was visually apparent. Because VSS and sensory assessments were performed shortly after healing rather than at a standardized interval, these outcomes are reported as exploratory short-term findings.

### 2.2. Surgical Technique

All fasciotomies were performed on an emergency basis under spinal anesthesia. Medially, a full-length skin incision was created 1 cm behind the medial tibial border, running from the tibial plateau to the medial malleolus; the superficial posterior compartment was then released at the anterior edge of the gastrocnemius, the gastrocnemius and soleus were retracted posteriorly, and the deep posterior compartment was decompressed. Laterally, a full-length incision was made along the fibula from the fibular head to the lateral malleolus, after which the anterior compartment and, over most of its length, the lateral compartment were released.

#### 2.2.1. SDT Group

After complete decompression, two 2-0 PDS sutures(PDS II; Ethicon, LLC, Somerville, NJ, USA) were placed on each side of the wound, drawing the edges from both flanks toward the midline, with needle spacing of 1.0–1.5 cm and a bite margin of 1.0 cm. After the initial stitch, the needle was passed through the suture loop to form a continuous locking pattern. The sutures were left untightened during the operation, and the paired ends were tied with a slipknot at the midline. The wound was dressed with povidone-iodine gauze and sterile cotton. Although PDS is absorbable, the sutures were removed about 2 weeks after wound closure to avoid leaving foreign material on the skin surface. PDS was chosen for its single-needle loop design, which allows the tail to be locked securely against slippage; its good tissue compatibility and maintained tensile strength during the distraction period; and its low-friction monofilament nature, which lessens discomfort during suture tightening (Figure 2).

#### 2.2.2. SG Group

After decompression, a vacuum-assisted closure (VAC) device with negative-pressure suction (closed negative-pressure irrigation and suction device, model MJ-03C4; Guangdong Meiji Biotechnology Co., Ltd., Guangzhou, Guangdong, China; closed negative-pressure wound dressing, PU-III; Guangzhou Rianhome Pharm & Tech Co., Ltd., Guangzhou, Guangdong, China) was applied to the wound. The VAC dressing was removed on postoperative day 7. Skin grafting was scheduled once healthy granulation tissue had formed; when granulation was insufficient, repeated debridement and further VAC therapy were undertaken until the wound bed became suitable. After the wound edges were approximated from both sides to diminish the defect, split-thickness skin grafts were harvested from the ipsilateral or contralateral thigh with a manual dermatome and placed as stamp grafts, followed by a VAC dressing over the grafted area.

### 2.3. Postoperative Management

Postoperatively, both groups received edema-reduction therapy with mannitol or sodium aescinate as indicated. In the SDT group, dressings were changed every 48 h beginning on postoperative day 2. At each change, the pre-positioned PDS sutures were cinched progressively from both sides toward the midline with two forceps, in accordance with the resolution of limb edema. Tension was adjusted to the patient’s pain tolerance and, in addition, was guided by bedside monitoring of skin perfusion, capillary refill, distal neurovascular status, wound-edge ischemia, and compartment symptoms; tightening was deferred or the sutures were loosened whenever impaired perfusion or excessive tension was suspected. In the SDT group, no suture cut-through, skin edge necrosis, blistering, wound dehiscence, recurrent compartment pressure, or failure to achieve closure was observed.

In most SDT patients, complete closure was achieved after 3–5 dressing changes with progressive tightening, and sutures were removed 2 weeks after closure. In the SG group, the VAC device was removed on postoperative day 7 to evaluate graft viability, after which serial dressing changes were continued until the wound healed completely.

### 2.4. Statistical Analysis

Statistical analysis was performed with SPSS version 27.0 (IBM Corp., Armonk, NY, USA). Because the decompression incisions were roughly spindle-shaped, wound area was approximated with the following formula: area = (diagonal length × width)/2; donor-site area was computed as length × width. The Shapiro–Wilk test was used to evaluate the normality of continuous variables. Normally distributed data are expressed as mean ± standard deviation and were compared between groups with the independent-samples Student’s *t*-test. Non-normally distributed data are expressed as median (interquartile range, Q1, Q3) and were compared with the Mann–Whitney U test. Categorical variables are summarized as frequencies and proportions and were compared using Pearson’s chi-square test or Fisher’s exact test, as appropriate. All tests were two-tailed, with the significance threshold set at *α* = 0.05. For the primary outcome (wound healing time), a parsimonious multivariable analysis was additionally performed to account for potential confounding. Because this outcome was positively skewed, it was log-transformed and entered into a multiple linear regression that included treatment group, time from injury to fasciotomy, and wound width as covariates; time from injury to fasciotomy was included because it was the only baseline variable that differed significantly between groups, and wound width was included as a clinically relevant correlate of closure difficulty. The adjusted treatment effect is reported as the exponentiated regression coefficient (adjusted ratio of wound healing times) with a 95% confidence interval (CI). Two robustness analyses were additionally performed to assess the stability of the primary effect estimate under alternative model specifications. First, the log-linear regression described above was repeated with additional adjustment for age. Second, a quantile (median) regression of wound healing time on treatment group, time from injury to fasciotomy, wound width, and age was fitted to estimate the adjusted median difference in healing time between groups, with a 95% confidence interval derived from the regression.

## 3. Results

Wound healing time was counted from the index fasciotomy to complete epithelialization of the wound, and the infection surveillance period ran from fasciotomy to the last outpatient visit. The number of operations was defined as the total number of operating-room procedures undertaken for wound management, including the index fasciotomy, secondary placement of distraction sutures, and skin grafting. Because both incisions in a given patient were treated with the same method, the analysis was performed at the patient level, and bilateral wound area was obtained by summing the two incisional areas. No missing data were encountered for the variables analyzed, and every patient was followed until wound healing and discharge. Wound healing time was predefined as the primary outcome, with all other endpoints considered secondary. Given the small sample and the low number of events, multivariable adjustment was not feasible for the binary secondary outcomes; for the continuous primary outcome (wound healing time), a parsimonious adjusted analysis was performed as described in the Statistical Analysis Section. Baseline characteristics are also reported to permit assessment of comparability between the groups. The de-identified patient-level dataset is provided in Table S1 (Supplementary Materials).

The study comprised 67 patients: 31 in the SDT group and 36 in the SG group. As shown in Table 1, the two groups did not differ significantly in age (*p* = 0.461), sex (*p* = 0.406), wound length (*p* = 0.462), wound width (*p* = 0.841), unilateral wound area (*p* = 0.649), bilateral wound area (*p* = 0.635), diabetes (*p* = 0.742), smoking (*p* = 0.406), open fracture (*p* = 1.000), or BMI (*p* = 0.638), indicating that most baseline features were broadly comparable. One exception deserves attention: the time from injury to fasciotomy was significantly longer in the SG group (median 5.7 h; interquartile range [IQR] 4.3–6.5) than in the SDT group (median 4.5 h; IQR 3.5–5.2; *p* = 0.012); this difference should be kept in mind when interpreting the between-group outcomes.

Compared with the SG group, the SDT group had significantly fewer operations (*p* < 0.001), shorter wound healing time (*p* < 0.001), shorter hospital stay (*p* < 0.001), and lower wound-closure-related costs (*p* < 0.001). Sensation remained normal in all SDT patients but was impaired in all SG patients (*p* < 0.001). VSS scores were significantly lower in the SDT group (*p* < 0.001), indicating more favorable scar appearance. Because a donor-site wound is an inherent and unavoidable consequence of skin grafting, no statistical comparison was performed for this endpoint; descriptively, none of the 31 SDT patients had a donor-site wound, whereas all 36 SG patients had one, and the two donor-site infections (both in the SG group) are included in the infection analysis above. The infection rate did not differ significantly between groups (*p* = 0.495). The Hodges–Lehmann location-shift estimate (SG minus SDT) was 5.0 days (95% CI, 4.0 to 6.0 days) for wound healing time (primary outcome), 1.0 operation (95% CI, 1.0 to 1.0) for the number of operations, 19.0 days (95% CI, 14.0 to 25.0 days) for hospital stay, and 12,450 CNY (95% CI, 11,040 to 14,306 CNY) for wound-closure-related costs. The comparative clinical outcomes are summarized in Table 2. In the primary adjusted analysis, the SG group had a significantly longer wound healing time, with an adjusted healing-time ratio of 1.412 (95% CI, 1.303 to 1.531; *p* < 0.001). To assess the robustness of the primary finding, wound healing time was additionally analyzed with adjustment for time from injury to fasciotomy, age and wound width. After adjustment, the between-group difference in wound healing time persisted (adjusted median difference, 5.2 days; 95% CI, 3.6 to 6.9 days; *p* < 0.001), with an adjusted healing-time ratio of 1.420 (95% CI, 1.311 to 1.539) for the SG relative to the SDT group in the log-linear model.

## 4. Discussion

Closing fasciotomy wounds after ACS remains a considerable clinical challenge, and the closure strategy may affect subsequent fracture care. Although skin grafting is an established reconstructive option, it carries well-recognized drawbacks—donor-site injury, sensory disturbance, hypertrophic scarring, higher costs, and longer hospitalization [2,3]. In recent years, various skin distraction devices and techniques have emerged as alternatives to grafting for fasciotomy wounds, yet each has its own limitations. SDT enables gradual approximation of the wound edges through repeated tightening at routine dressing changes, allowing the incision to close progressively as edema subsides and providing a more continuous and efficient closure course.

### 4.1. Clinical Outcomes of the Suture Distraction Technique

In this cohort, the SDT group achieved better short-term results across several key clinical parameters than the SG group (Table 2). With respect to the number of operations, SG patients required at least two procedures—fasciotomy with VAC coverage followed by skin grafting—and some needed three operations because of inadequate granulation tissue. In contrast, SDT patients who had primary suture placement received the distraction apparatus during the index fasciotomy, avoiding an additional surgical procedure. It should be stressed that part of this difference is inherent to the two treatment protocols: all SG patients underwent VAC therapy followed by a planned grafting operation, whereas most SDT sutures were placed during the index fasciotomy. The observed difference therefore reflects a comparison of two complete treatment strategies rather than a biological advantage of SDT per se.

For the primary outcome, wound healing time was shorter in the SDT group (median 14.0 days) than in the SG group (median 18.5 days) (*p* < 0.001; Hodges–Lehmann location-shift estimate [SG minus SDT], 5.0 days; 95% CI, 4.0 to 6.0 days). The adjusted analysis supported these findings: after controlling for time from injury to fasciotomy and wound width, the SG group still had a significantly longer healing time (adjusted ratio 1.412; 95% CI 1.303–1.531; *p* < 0.001), indicating that the advantage of SDT was not explained by the between-group imbalance in time from injury to fasciotomy. This advantage persisted in robustness analyses with further adjustment for age (adjusted ratio 1.420; 95% CI 1.311–1.539; *p* < 0.001). Median hospital stay was 27.0 days in the SDT group versus 46.0 days in the SG group (*p* < 0.001), representing a difference of 19 days between the reported medians, consistent with the review by Guo et al. [12]. Nevertheless, hospital stay is influenced not only by wound closure but also by fracture treatment and associated injuries, and the between-group difference cannot be ascribed solely to the closure method.

Regarding costs, the median wound-closure-related expenditure was 1656 CNY in the SDT group versus 14,926.5 CNY in the SG group (*p* < 0.001). Among the 27 SDT patients who underwent primary intraoperative suture placement, the charge was 1656 CNY covering PDS suture material and operative fees in 26 of the 27 patients, whereas the remaining patient incurred 1305 CNY; the four patients treated with secondary suture placement incurred additional operative costs (range of 5376–14,627 CNY). The SG group, by contrast, faced markedly higher expenditures driven largely by VAC consumables and grafting procedures, suggesting a substantial cost advantage for SDT. Costs were retrieved from the hospital billing system at the price level of the respective admission and were not adjusted for inflation over the seven-year study period. Moreover, because enrollment spanned more than seven years, temporal changes in pricing or clinical practice cannot be excluded; the two treatment groups were not matched on enrollment period, and the cost comparison should therefore be interpreted with this caveat. With respect to functional and esthetic outcomes, the SDT group fared favorably: all SDT patients preserved normal sensation, whereas all SG patients had hypoesthesia of varying severity at both the graft and donor sites (*p* < 0.001), which is most plausibly attributable to transection of cutaneous nerve endings during graft harvest. Scars after SDT were smaller and more cosmetically acceptable, in line with the observations of Fang et al. [13]. The VSS score of 0 recorded in most SDT patients reflects evaluation at the final outpatient visit shortly after healing; given the short follow-up, these scores represent early scar status and do not preclude subsequent scar remodeling. Accordingly, both VSS and sensory results should be regarded as exploratory short-term outcomes. Of note, “secondary donor-site injury” is a structurally predetermined endpoint, since every grafted patient necessarily has a donor wound whereas every SDT patient does not; donor-site pain, delayed healing, infection, hypertrophic scarring, pigmentation, and sensory deficit are the clinically meaningful outcomes and warrant separate evaluation in future studies.

Regarding safety, the infection rate was comparable between groups (0/31 vs. 2/36; *p* = 0.495). Because the number of events was small, however, this comparison is underpowered and should be regarded as inconclusive rather than as proof of equivalent safety; a larger cohort is needed to rule out a clinically meaningful difference in infection risk.

### 4.2. Advantages of the Suture Distraction Technique

Fasciotomy wounds in ACS constitute a distinct category of wounds in which true skin loss is absent [11]. Under slow, sustained tensile stress, SDT expands the available skin through two mechanisms: mechanical creep and biological growth [14]. Whether applied primarily at the index operation or as delayed secondary closure, PDS suture distraction achieves reliable wound closure. Critically, the decompression incision in ACS is not associated with substantive skin loss: the skin is divided surgically rather than destroyed by trauma, and the wound edges remain viable and can be re-approximated once compartment pressure is relieved. This wound type may thus be particularly suited to SDT, which permitted complete closure without grafting in all SDT patients in this cohort, sparing them secondary surgical injury, additional hospitalization costs, and longer stays. Moreover, our earlier report [11] dealt with wound classification and lower-limb skin defects in general, whereas the present study addresses ACS decompression incisions specifically—a distinct wound type without skin loss—and evaluates wound-closure-related outcomes; this distinction defines the research question and incremental contribution of the present work.

### 4.3. Comparison with Conventional Dermatotraction Techniques

Relative to commercial skin distraction systems, such as the DermaClose device [15], TopClosure system [16], external fixation-based traction devices [17], and bidirectional adjustable skin closure systems [18], PDS sutures offer practical advantages as absorbable monofilament suture material with excellent tissue biocompatibility and minimal tissue reactivity, properties that suit wounds requiring sustained distraction. SDT needs no dedicated equipment, entails negligible material costs, and is easy to perform with straightforward postoperative care. In contrast, the commercial devices cited above are comparatively costly, as Song et al. [9] and Zhang et al. [19] also noted in their studies of modified skin distraction devices.

Compared with the established shoelace closure and vessel-loop approaches [20,21,22,23], SDT offers four main advantages: (1) the procedure is technically simple and can be performed at the bedside, tolerates considerably greater tensile loads without suture pull-out, and avoids the need for further suturing once the edges are approximated; (2) two continuous locking PDS sutures placed bilaterally at the index fasciotomy distribute tension evenly along the full length of the wound; (3) a midline slipknot enables progressive, stepwise tightening during bedside dressing changes; and (4) a standardized timing protocol starts distraction immediately after decompression and continues it as limb edema resolves. Together, these features distinguish SDT from earlier gradual approximation techniques.

### 4.4. Limitations

Several limitations deserve mention. First, this retrospective, single-center study with a modest sample (*n* = 67) is prone to selection and temporal bias. Second, the distraction rate could not be quantified precisely, because tension was titrated against patient pain tolerance; sensation was evaluated globally rather than at anatomically matched sites; the short follow-up limited long-term functional and scar assessment; and analgesia use and discomfort during bedside tightening were not captured. In addition, costs were not adjusted for inflation, and the calendar-year distribution of the two techniques over the seven-year enrollment period could not be fully standardized. Third, the conclusions apply only to lower-leg ACS; as Liu et al. [24] and Sraj et al. [25] observed, the methods and timing of fasciotomy wound closure vary widely, underscoring the need for standardized protocols and higher-level evidence. Multicenter prospective studies with larger cohorts and longer sensory and scar follow-up are thus warranted.

## 5. Conclusions

SDT is a simple, inexpensive approach that relies solely on PDS sutures for progressive closure of ACS decompression incisions. In this selected single-center retrospective cohort, SDT was associated with fewer operations, shorter wound healing time and hospital stay, lower costs, better scar appearance, and preserved sensation, thereby avoiding donor-site morbidity. Because the decompression incision involves no substantive skin loss, it may be a strong candidate indication for SDT, which allowed complete wound closure without grafting in this series. Although the distraction rate could not be quantified precisely, these findings merit confirmation through prospective comparative studies.

## Figures and Tables

**Table 1:** Baseline characteristics of the two groups.

Variable	SDT Group (*n* = 31)	SG Group (*n* = 36)	*p*-Value
Age (years)	44.39 ± 15.35	41.69 ± 14.32	0.461
Sex, male, *n* (%)	17 (54.8%)	15 (41.7%)	0.406
Wound length (cm)	22.0 (20.0, 23.5)	22.0 (20.0, 24.25)	0.462
Wound width (cm)	7.23 ± 1.71	7.31 ± 1.53	0.841
Unilateral wound area (cm^2^)	79.21 ± 28.29	82.21 ± 25.42	0.649
Bilateral wound area (cm^2^)	157.35 ± 46.63	152.38 ± 38.81	0.635
Concomitant diabetes, *n* (%)	4 (12.9%)	6 (16.7%)	0.742
Smoking, *n* (%)	17 (54.8%)	15 (41.7%)	0.406
Open fracture, *n* (%)	8 (25.8%)	10 (27.8%)	1.000
Time from injury to fasciotomy (h)	4.5 (3.5, 5.2)	5.7 (4.3, 6.5)	0.012
BMI (kg/m^2^)	23.85 ± 2.88	24.20 ± 3.20	0.638

SDT, suture distraction technique; SG, skin grafting. Continuous variables are presented as mean ± standard deviation or median (interquartile range, Q1, Q3). Categorical variables are presented as frequencies and percentages. Unilateral wound area refers to the area of the first (lateral) incision in patients with dual incisions, and it likewise refers to the (lateral) incision in the single patient who had only one decompression incision; bilateral wound area represents the sum of the two incisional areas in patients with dual incisions, calculated from the final dataset.

**Table 2:** Comparison of clinical outcomes between the two groups.

Outcome Measure	SDT Group (*n*= 31)	SG Group (*n*= 36)	Difference (95% CI)	*p*-Value
Number of operations	1.0 (1.0, 1.0)	2.0 (2.0, 2.0)	1.0 (1.0 to 1.0)	<0.001
Number of suture tightening sessions	3.0 (3.0, 3.5)	—	—	—
Wound healing time (days)	14.0 (12.0, 15.0)	18.5 (17.0, 20.0)	5.0 (4.0 to 6.0)	<0.001
Length of hospital stay (days)	27.0 (22.5, 32.5)	46.0 (42.75, 58.5)	19.0 (14.0 to 25.0)	<0.001
Wound-closure-related costs (CNY)	1656.0 (1656.0, 1656.0)	14,926.5 (11,971.5, 16,631.25)	12,450 (11,040 to 14,306)	<0.001
Hypoesthesia, *n* (%)	0 (0.0%)	36 (100.0%)	—	<0.001
Donor-site wound (inherent), *n* (%)	0 (0.0%)	36 (100.0%)	—	—
Donor-site length (cm)	0	14.56 ± 2.06	—	—
Donor-site width (cm)	0	9.39 ± 2.41	—	—
Donor-site area (cm^2^)	0	137.50 ± 41.77	—	—
Infection, *n* (%)	0 (0.0%)	2 (5.6%)	—	0.495
VSS score (points)	0.0 (0.0, 1.0)	4.0 (4.0, 5.0)	4.0 (4.0 to 5.0)	<0.001

SDT, suture distraction technique; SG, skin grafting; VSS, Vancouver Scar Scale; CNY, Chinese Yuan; CI, confidence interval. Continuous variables are presented as median (interquartile range, Q1, Q3) or mean ± standard deviation, and categorical variables as frequencies and percentages. Between-group differences (SG minus SDT) and their 95% CIs were estimated using the Hodges–Lehmann location-shift estimator (continuous variables); positive values denote higher values in the SG group, i.e., the magnitude by which outcomes favored the SDT group. Differences are not reported for structurally predetermined endpoints or for variables measured in a single group.

## Data Availability

The de-identified dataset supporting the findings of this study is available as Supplementary Material, including patient demographics, injury and treatment characteristics, wound healing parameters, hospital stay, and costs. All personally identifiable information has been removed. Further inquiries can be directed to the corresponding author.

## Supplementary Materials

The following supporting information can be downloaded at https://www.mdpi.com/article/10.3390/jcm15207971/s1. Table S1: De-identified patient-level dataset, including patient demographics, injury and treatment characteristics, wound healing parameters, hospital stay, and costs.
